# Tantalum-Doped Co–Cr Alloys: Multiscale Effects on Mechanics, Surface Potential, and Biological Responses

**DOI:** 10.1021/acsomega.6c02438

**Published:** 2026-07-15

**Authors:** Beatriz da Silva Batista, Rosa Maria Viana Sousa, Samuel Filgueiras Rodrigues, Napoleão Martins Argôlo Neto, Alan Silva de Menezes, Luzeli Moreira da Silva, Pierre Basílio Almeida Fechine, Ralph Santos-Oliveira, Luciana Magalhães Rebêlo Alencar

**Affiliations:** † Center for Social Sciences, Health and Technology, 37892 Federal University of Maranhão, Advanced Unit, Imperatriz, Maranhão 65915-060, Brazil; ‡ Department of Physics, Laboratory of Biophysics and Nanosystems, Federal University of Maranhão, Campus Bacanga, São Luís, Maranhão 65085-580, Brazil; § Federal Institute of Education, Science and Technology of Maranhão, Monte Castelo, São Luís, Maranhão 65030-005, Brazil; ∥ Postgraduate Program in Technologies Applied to Animals of Regional Interest, Federal University of Piauí, Teresina, Piauí 64049-550, Brazil; ⊥ Federal University of Piauí, Bachelor of Veterinary Medicine, Teresina, Piauí 64049-550, Brazil; # Integrated Center for Morphology and Stem Cell Research, Federal University of Piauí, Teresina, Piauí 64049-550, Brazil; ∇ Advanced Materials Chemistry Group, Department of Analytical Chemistry and Physical Chemistry, Federal University of Ceará, Campus do Pici, Fortaleza, Ceará 60455-760, Brazil; ○ Brazilian Nuclear Energy Commission, Institute of Nuclear Engineering, 54531 Laboratory of Nanoradiopharmacy and Synthesis of New Radiopharmaceuticals, Rio de Janeiro 21941-906, Brazil; ◆ State University of Rio de Janeiro, Laboratory of Radiopharmacy and Nanoradiopharmaceuticals, Rio de Janeiro 23070-200, Brazil

## Abstract

Cobalt–chromium (Co–Cr) alloys are valued in biomedicine for their mechanical performance and relative bioinertness. Here, Co–Cr alloys containing tantalum (Ta; 0, 3, 6, and 9 wt %) were synthesized by electric-arc melting to assess how Ta content influences bulk and surface properties across scales. X-ray Diffraction (XRD), Vickers Microhardness, Wettability, Scanning Electron Microscopy (SEM), Energy-Dispersive X-ray Spectroscopy (EDS), and Atomic Force Microscopy/Kelvin Probe Force Microscopy (AFM/KPFM) were employed. Bioactivity and biocompatibility were evaluated by immersion in simulated body fluid (SBF) and cell-viability assays. Ta addition promoted the coexistence of Co allotropes (εCo and αCo) and the formation of a secondary TaCo_2_ phase, resulting in a dendritic microstructure. Microhardness increased significantly, reaching 466 ± 27 HV0.5 for 9 wt % Ta (≈77% above the Ta-free alloy). Surface-potential mapping revealed ≈1500% higher local potential differences and composition-dependent work function distribution, indicating Ta-driven electronic heterogeneity. The alloys were cytocompatible under extract exposure conditions, suggesting that Ta-induced surface changes may indirectly modulate the biological response. SBF tests confirmed improved *in vitro* bioactivity, with apatite nucleation on all alloys; the 6 wt % Ta composition produced the thickest Ca/P-rich layer (∼1.3 μm). Overall, Ta addition modulated the microstructure, localized deformation resistance, surface electronic response, and *in vitro* biological behavior of Co–Cr alloys, with 6 wt % Ta showing the most balanced response among the investigated compositions.

## Graphical abstract

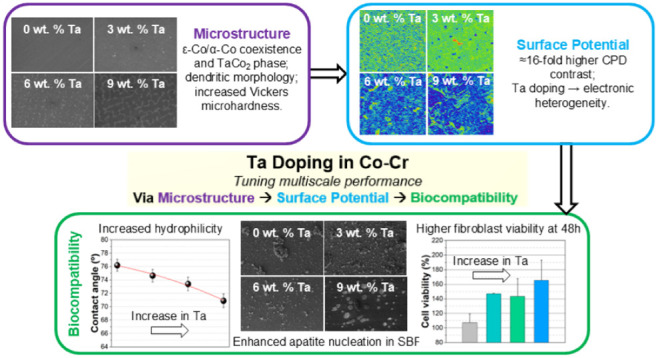

## 1 Introduction

The need for more robust, cytocompatible metallic biomaterials led to the development of alloys with superior properties for medical applications. Cobalt–chromium (Co–Cr) alloys are widely employed in orthopedic implants and dental prosthetic components due to their high strength, hardness, and corrosion and wear resistance.^1,2^ In addition to this favorable balance of properties, Co–Cr alloys exhibit superior biocompatibility, which supports their widespread clinical adoption.^3^ Elemental modification, particularly with tantalum (Ta), has emerged as a strategy to further customize performance by influencing microstructure, passivity, and tribocorrosion behavior.^4^ Ta is noted for its excellent biocompatibility and corrosion resistance^5,6^ and can improve the performance of Co–Cr alloys, expanding their usefulness as implant materials. Moreover, controlled alloying can alter surface electronic properties, such as surface potential. These properties are key determinants of the biomaterial-tissue interface and early cell responses.^7,8^

Surface potential and work function are key descriptors for understanding how metallic alloys behave in biological environments. Surface potential governs electrostatic behavior at the interface, influencing the adsorption of proteins and ions, as well as early cell-material interactions.^9–11^ By contrast, the work function is the minimum energy required to remove an electron to a vacuum; in ambient or liquid conditions, it reflects the Fermi level and can modulate interfacial charge-transfer processes and adsorption energies.^12^ These properties are linked to crystal structure, passive-film chemistry, corrosion resistance, and hardness, parameters that are critical to optimizing metallic alloys for use as biomaterials.^13^ For Co–Cr stent alloys, controlled modifications of the surface oxide altered both the amount and the conformation/profile of adsorbed blood proteins (e.g., fibrinogen and albumin), thereby affecting early biological activation at the implant interface.^14,15^ Similarly, Ta-containing metallic surfaces exhibit accelerated apatite formation in simulated body fluid (SBF), attributed to reactive Ta–OH groups that promote calcium-phosphate nucleation.^15^ At the nanoscale, atomic force microscopy with Kelvin probe force microscopy (AFM/KPFM) has been used to map surface-potential variations, revealing local electronic heterogeneities that can influence protein adsorption and subsequent cell behavior.^9^

KPFM is an AFM-based technique that maps the surface potential at nanometer-scale spatial resolution. A conductive probe is driven with an AC bias while a DC nulling bias is applied to cancel the electrostatic force (or its gradient) between tip and sample; the resulting DC value yields the contact potential difference (CPD).^16^ Under proper calibration of the tip work function (*φ*_tip_) against a reference, CPD maps can be converted into relative work function maps of the surface. By resolving local variations in surface potential/work function, KPFM reveals electronic heterogeneities associated with compositional changes, doping, treatments, or structural modifications. These nanoscale characteristics can modulate interfacial charge transfer, ion/protein adsorption, and early cell-material interactions, providing mechanistic insight into how processing alters the electrical landscape of metallic surfaces.^17,18^

Previous studies^4,19^ reported that adding 9 wt % Ta to Co–Cr alloys modify microstructure and surface potential while delivering corrosion rates compatible with *in vivo* use. Based on this argument, this work investigated intermediate Ta contents (3 and 6 wt %) to elucidate composition-property relationships across the Co72-xCr28Tax series (x = 0, 3, 6, 9; wt %). Here, we mapped surface potential by KPFM as CPD and, when appropriately calibrated, inferred relative work function variations. These electrical characteristics were correlated with crystal structure, wettability, bioactivity, and cellular response to clarify how Ta doping influences the multiscale performance of Co–Cr–Ta biomaterials.

## 2 Methodology

### 2.1 Synthesis and Surface Preparation of the Samples

The Co_72–x_Cr_28_Ta_*x*_ alloys (x = 0, 3, 6, and 9 wt %) were obtained by arc furnace melting with high-purity elements (Co: 99.5%, Cr: 99.9%, Ta: 99.5%; Sigma-Aldrich) weighed in stoichiometric proportions. During the melting process, the alloys were turned and remelted three times to ensure good homogeneity. After melting, the cooled alloys were sealed in quartz tubes with an inert argon atmosphere. They were then subjected to heat treatment in a muffle furnace (Jung, J200), heating them to 1000 °C at a rate of 5 °C/min and maintaining this temperature for 48 h, followed by rapid cooling. After heat treatment, the alloys were cut using a cutting machine to obtain the samples in slice form. Before all characterizations, the samples underwent surface preparation by mechanical polishing with SiC sandpaper up to 3000 grits, followed by polishing with diamond paste to 0.25 μm. Subsequently, the samples were cleaned in an ultrasonic bath containing acetone, isopropyl alcohol, and distilled water for 10 min each, and subsequently dried under clean air. This protocol was used for all samples to minimize differences associated with polishing residues and surface contamination. The samples are appointed in work according to their composition: Co_72_Cr_28_ (0 wt % Ta), Co_69_Cr_28_Ta_3_ (3 wt % Ta), Co_66_Cr_28_Ta_6_ (6 wt % Ta), and Co_63_Cr_28_Ta_9_ (9 wt % Ta).

### 2.2 Wettability

The surface wettability of the samples was calculated from the contact angle measurement using an advanced sessile drop method, the DropSnake drop shape analysis (active contours).^20^ A 3 μL drop of distilled water was deposited with a micropipette perpendicularly to the surface of the samples, and the drop profile was recorded after 3 min using a USB digital microscope. From the generated images, the contact angle was measured using the DropSnake method with the aid of ImageJ software (NIH, v. 1.53k).^21^ Ten measurements were performed for each sample, with drops randomly positioned on the surface. Measurements were performed at controlled room conditions (25 °C, 45% RH).

### 2.3 Calcium Phosphate Growth

Calcium-phosphate deposition was evaluated *in vitro* by immersion of polished samples in simulated body fluid (SBF), prepared according to the protocol established by Kokubo et al.^22^ Specimens were incubated in SBF for 28 days in a temperature-controlled water bath at 36.5 °C, and the pH was measured daily. After immersion, samples were gently rinsed with deionized water to remove residual salts and then air-dried at ambient temperature (25 °C) before subsequent characterization.

### 2.4 Cell Viability

Cytocompatibility was assessed by exposure of murine fibroblasts (L929, ATCC CCL-1) to the alloy extracts, followed by MTT-based viability quantification, in accordance with ISO 10993-5.^23^ Disc-shaped samples, 0.9 cm in diameter and 0.1 cm thick, corresponding to a total exposed area of approximately 1.56 cm^2^, were used for extract preparation. Before extraction, the polished samples were cleaned by sequential ultrasound in acetone, isopropyl alcohol, and sterile distilled water for 10 min each, followed by drying under sterile airflow. The samples were then transferred to a biological safety cabinet and immersed in 70% ethanol for 30 min. After ethanol exposure, the discs were rinsed three times with sterile ultrapure water to remove residual ethanol. The samples were then exposed to ultraviolet light for 30 min on each side, under aseptic conditions, and immediately used for extract preparation.

The L929 line was selected because it is widely adopted as a reference cell line for screening the cytotoxic potential of medical-device materials, including metallic alloys.^24,25^ Fibroblasts were cultured in high-glucose DMEM (Capricorn, ref 150763) supplemented with 10% fetal bovine serum (BNB, ref 150023) and 1% penicillin-streptomycin (Capricorn, ref 150772) at 37 °C, 5% CO_2_, and 95% relative humidity. Negative controls consisted of cells cultured in supplemented medium, while positive controls consisted of cells cultured in supplemented medium containing 10% DMSO. For each alloy composition, three independent sterilized alloy specimens were individually immersed in 1 mL of supplemented culture medium and incubated for 24 h at 37 °C and 5% CO_2_. Thus, three independently prepared alloy extracts were obtained for each composition. Each extract was transferred to a separate well containing L929 fibroblasts and was evaluated under the same exposure conditions for 24 and 48 h. Therefore, the three replicate measurements represent independent specimen-level extract replicates derived from three separate alloy specimens. Results are reported as mean ± standard deviation of the three independently prepared extracts.

After each exposure period, the culture medium was removed, the wells were washed with phosphate-buffered saline (PBS), and 100 μL of MTT solution (0.5 mg/mL) was added to each well. After 4 h of incubation, the solution was removed, and the resulting formazan crystals were dissolved in 100 μL of DMSO. Absorbance was measured at 570 nm using a microplate reader (BioTek ELx800). Cell viability was calculated using eq 1, where A_t_ represents the absorbance of the treatment groups and A_c_ represents the absorbance of the negative control group. The MTT result corresponds to cells cultured on the plate and exposed to extracts independently prepared from three separate alloy specimens per composition, rather than to cells directly attached to the alloy surfaces.

1 $$Cell\;viability=\frac{A_{t}}{A_{c}}\times 100$$

### 2.5 X-ray Diffraction

The X-ray diffraction (XRD) patterns of the metal alloys were obtained on bulk samples at room temperature. A diffractometer (D8 Advance, Bruker) with Cu Kα radiation (λ = 1.5406 Å), a voltage of 40 kV, and a current of 40 mA was used to obtain the diffractogram in the range (2θ) from 20 to 100°, with an angular step of 0.02° and a counting time of 1 s per step. The measurements were performed at four azimuthal angles, 0, 40, 90 and 140°. From the obtained diffractograms, the sample exhibiting the highest number of crystallographic peaks was selected for further analysis.

### 2.6 Vickers Microhardness

Resistance to localized plastic deformation was evaluated by Vickers microhardness on polished surfaces using a microhardness tester (Shimadzu HMV-2T) with a square-based pyramidal diamond indenter, in accordance with ISO 6507-1 (and consistent with ASTM E384). For each composition, n = 10 indentations were performed with a 0.5 kgf load (HV0.5; 4.903 N) and 15 s dwell time. Indents were placed with a center-to-center spacing of ≥3× the measured indentation diagonal and ≥2.5× the diagonal from sample edges to avoid interaction and edge effects; the indenter axis was normal to the sample surface. Hardness values were reported as mean ± SD. Group differences were assessed by one-way ANOVA followed by Tukey’s post hoc test, with *p* ≤ 0.05 considered statistically significant.

### 2.7 Scanning Electron Microscopy and Energy Dispersive X-ray Spectroscopy

The surface morphology of the alloys was examined by scanning electron microscopy (SEM; Zeiss EVO) using a secondary-electron (SE) detector at magnifications up to 12,000×. Unless otherwise stated, images were acquired on polished, unetched surfaces. Areal fractions of contrast-distinct regions were estimated from SEM micrographs by grayscale thresholding in ImageJ (NIH, v1.53k), reporting the areal fraction relative to the analyzed field of view. We note that such image-based segmentation reflects 2D contrast (topographic/electronic/compositional) and is therefore an approximation to phase fraction. Complementary chemical analysis was performed by energy-dispersive X-ray spectroscopy (EDS; Bruker XFlash 410M). Spectra/maps were acquired at 15–20 kV accelerating voltage on polished areas to determine elemental composition.

### 2.8 Atomic Force Microscopy

Data was acquired with an Atomic Force Microscope (AFM, Bruker, Multimode 8) equipped with a NanoScope V controller, using the Kelvin Probe Force Microscopy (KPFM) mode to obtain topography and detect the surface potential and respective work function. Before KPFM measurements, all alloy samples were prepared using the same surface-treatment protocol as previously described in Section 2.1. KPFM measurements were performed under controlled ambient conditions, 25 °C and 45% RH, using the same conductive probe (Bruker, PFQNE-AL, k = 0.8 N/m) and the same acquisition parameters for all samples (resolution of 256 × 256 pixels and a scan rate of 0.5 Hz). Topography was first acquired, and the surface potential was then measured in lift mode using a lift height of 70 nm to reduce short-range topographic contributions to the electrostatic signal. Regions containing obvious polishing debris or topographic defects were avoided for quantitative analysis whenever possible. The potential maps were interpreted only when the contrast was not spatially correlated with isolated topographic particles and when it followed the microstructural contrast observed by SEM/EDS.

The tip work function was calibrated using an Al–Si–Au reference grid and an independently characterized conductive probe. Details of the calibration procedure and conversion of surface potential data into work function values are provided in the Supporting Information (Figures S1–S4). The resulting work function values obtained for the reference materials showed good agreement with literature data,^26,27^ supporting the validity of the adopted methodology. The Al–Si–Au reference was measured before and after the sample scans to evaluate instrumental stability. Therefore, the work-function data are discussed primarily in terms of relative differences and distribution trends among samples measured under identical conditions. Reported work-function values are given as medians across the mapped area; in multimodal cases, Gaussian mixture fits were used to identify subpopulations. Data preprocessing (polynomial background removal and line alignment) was performed using Gwyddion software (v. 2.63).

## 3 Results

### 3.1 Structural, Morphological, and Hardness Properties

XRD analysis (Figure 1a) revealed that the chemical composition and cooling conditions promoted the formation of a heterogeneous system comprising three distinct phases: (1) hexagonal compact (space group *P*6_3_/*mmc*, ICSD#44990),^28^ *ε*Co; (2) face-centered cubic (space group *Fm*3̅*m*, ICSD#53805),^29^ *α*Co; and (3) hexagonal TaCo_2_ (space group *P*6_3_/*mmc*, ICSD#625333).^30^ The TaCo_2_ phase emerged in the Co_66_Cr_28_Ta_6_ alloy, as evidenced by new diffraction peaks, with peak intensities progressively increasing with higher Ta content (Co_63_Cr_28_Ta_9_), indicating greater phase abundance. The resistance to localized plastic deformation for the series of samples was obtained by Vickers microhardness (Figure 1b). It can be observed that the microhardness increases as Ta is inserted into the Co–Cr matrix, with mean values and standard deviations of 263 ± 20 (Co_72_Cr_28_), 305 ± 18 (Co_69_Cr_28_Ta_3_), 353 ± 19 (Co_66_Cr_28_Ta_6_), and 466 ± 27 HV0.5 (Co_63_Cr_28_Ta_9_). The behavior of this property follows a polynomial function (y = 227 + 5x + 2x^2^, R^2^ = 0.99) for hardness as a function of Ta concentration.

**Figure 1:**
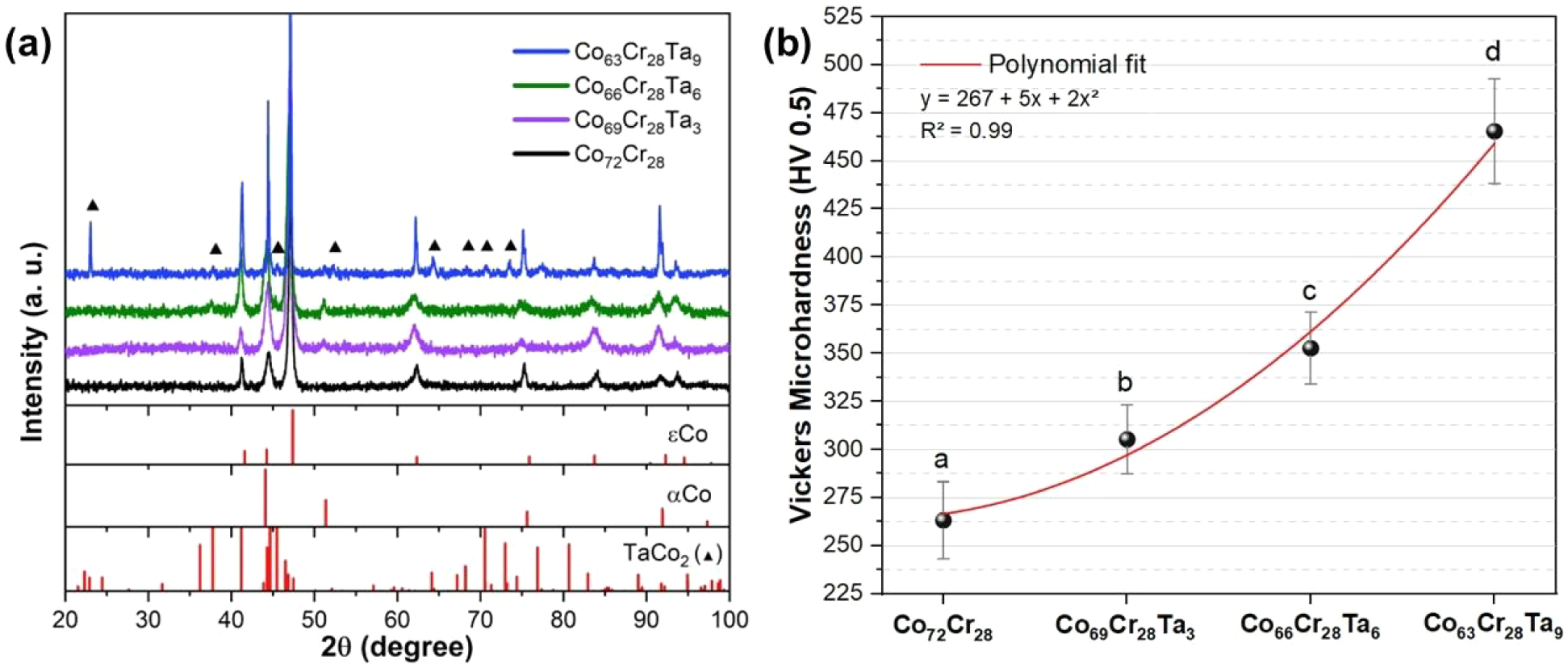
(a) X-ray diffraction patterns of the polished samples: the bar graphs represent the crystallographic phases εCo, αCo, and TaCo_2_ present in the samples. The triangle symbol represents the peaks arising from the TaCo_2_ phase. (b) Resistance to localized plastic deformation by Vickers microhardness: The spheres indicate the mean value, and the red line indicates the polynomial fit, while the bars are the standard deviations for the samples. Means followed by the same lowercase letter do not differ from each other by the Tukey test (*p* < 0.05).

SEM analysis (Figure 2) reveals a pronounced variation in contrast in the microstructure resulting from incorporating Ta into the Co–Cr matrix. EDS mapping and spot analysis (Figure 2) demonstrate that the high-contrast regions correspond to the elements Ta and Co (identified as TaCo_2_), while the low-contrast areas represent the Cr-enriched Co–Cr-based matrix (αCo and εCo) with lower Ta solubility. Quantitative image analysis using ImageJ software showed that the fraction of the TaCo_2_ phase increases substantially with Ta content, evolving from 1.3 ± 0.4% in Co_69_Cr_28_Ta_3_ to 3.7 ± 0.4% in Co_66_Cr_28_Ta_6_ and reaching 13.0 ± 0.7% in Co_63_Cr_28_Ta_9_.

**Figure 2:**
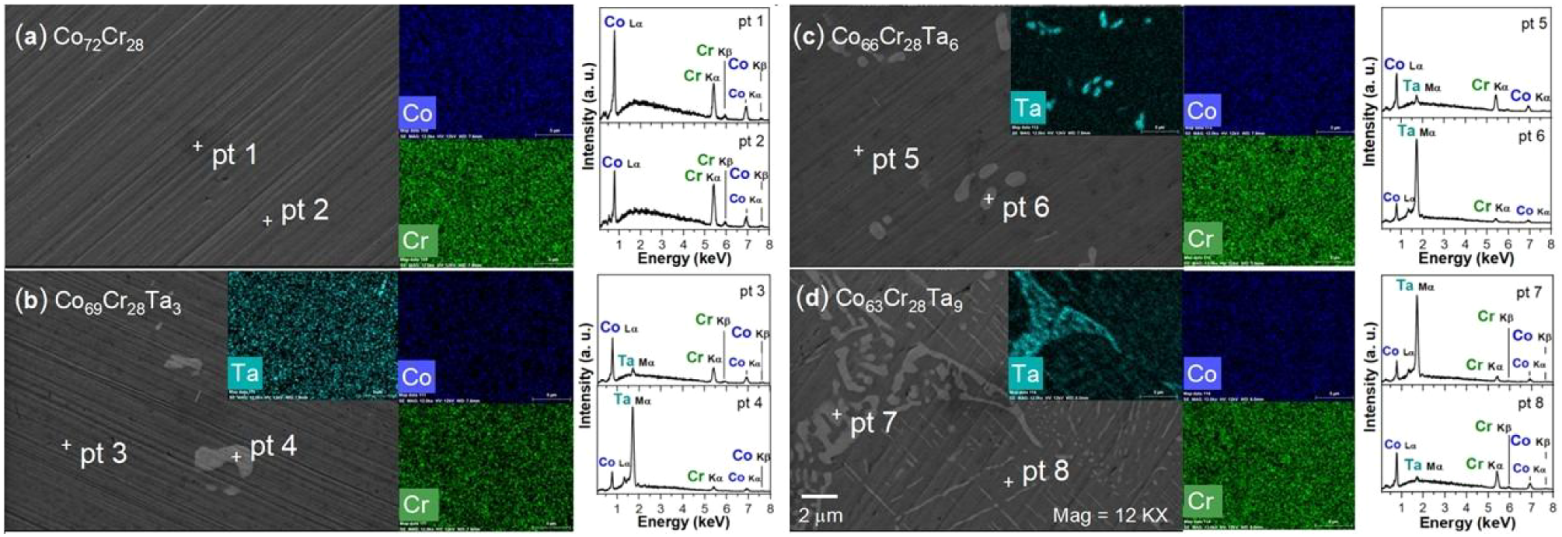
Surface morphological and elemental information (12 KX magnification): surface microstructure image, compositional maps, and EDS-described points 1–8 of (a) Co_72_Cr_28_, (b) Co_69_Cr_28_Ta_3_, (c) Co_66_Cr_28_Ta_6_, and (d) Co_63_Cr_28_Ta_9_.

### 3.2 Potential and Work Function Data by KPFM

The potential distribution on the sample surfaces was obtained by KPFM (Figure 3). The height maps (Figure 3a–d) reveal nanometric-scale surfaces with the presence of particles of the paste used in polishing, while the potential maps (Figure 3e–h) show that the distribution is not related to the topographies of the samples but rather to the morphology initially identified by SEM (Figure 2). Because KPFM measurements on metallic biomaterials are sensitive to the native/passive oxide layer, adsorbates, humidity, and surface preparation, the work-function values reported here should be interpreted as effective surface work-function responses of polished and air-exposed alloys, rather than as intrinsic work functions of bare metallic phases. Nevertheless, all samples were prepared and measured under identical conditions, and the systematic spatial correspondence between SEM/EDS microstructural contrast and KPFM potential contrast supports the interpretation that the observed electronic heterogeneity is mainly associated with Ta-induced microstructural heterogeneity, particularly the coexistence of Co–Cr-rich matrix regions and TaCo_2_-rich precipitates.

**Figure 3:**
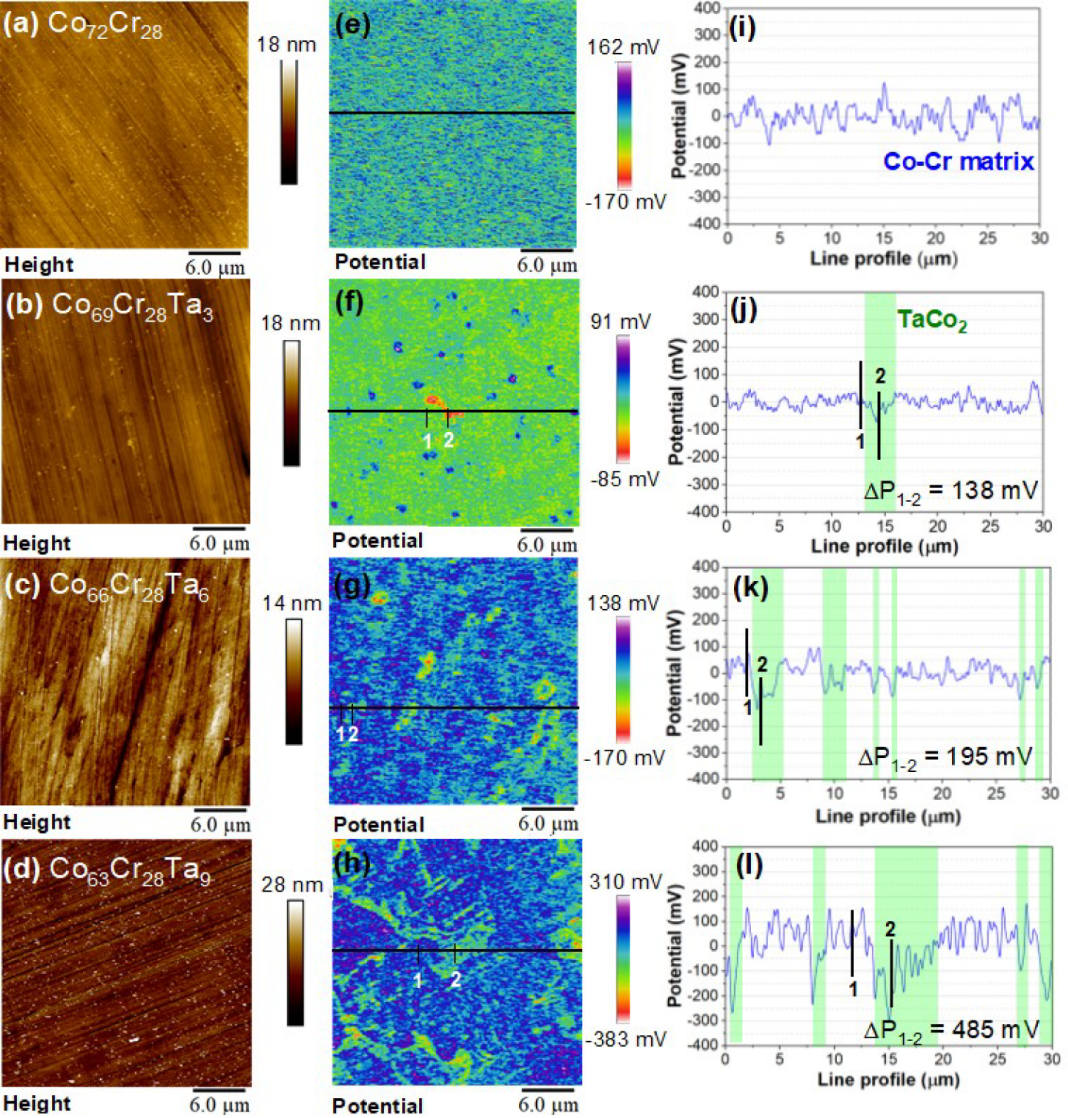
Topography, potential, and line profile by KPFM: (a, e, i) Co_72_Cr_28_, (b, f, j) Co_69_Cr_28_Ta_3_, (c, g, k) Co_66_Cr_28_Ta_6_, and (d, h, l) Co_63_Cr_28_Ta_9_.

The positions of the line profiles (Figure 3i–l) indicate the regions with the presence of the TaCo_2_ phase and the relative potential difference (ΔP_1–2_) between these regions and the Co–Cr matrix. For the Ta-containing alloys, ΔP was calculated as the local contact potential difference between Co–Cr-rich matrix regions and TaCo_2_-rich regions along KPFM line profiles. For the Co_72_Cr_28_ alloy, which does not contain TaCo_2_, ΔP was obtained from different positions along line profiles on the Co–Cr matrix and represents the baseline local CPD variation of the Ta-free alloy, rather than an interfacial potential difference between distinct phases.

It can be observed that the relative potential differences (ΔP_1–2_) between the TaCo_2_ phase and the Co–Cr matrix measured for the maps in Figure 3 increase as Ta precipitates in the structure. These differences correspond to regions characterized by the segregation of the chemical elements of the alloy. To understand the trend of these data along the sample surfaces, the relative potential differences (ΔP_1–2_) between the Co–Cr matrix and the TaCo_2_ phase were measured in 30 different areas for each sample, obtaining an average value ± standard deviations presented in Figure 4a. It can be seen that ΔP increased as Ta was inserted into the Co–Cr matrix, with average values and standard deviations of 43 ± 27 (Co_72_Cr_28_), 99 ± 31 (Co_69_Cr_28_Ta_3_), 217 ± 59 (Co_66_Cr_28_Ta_6_), and 687 ± 73 mV (Co_63_Cr_28_Ta_9_). The behavior of this property obeys a polynomial function (y = 48 – 17x + 9x^2^, R^2^ = 0.98) for ΔP as a function of the Ta concentration.

**Figure 4:**
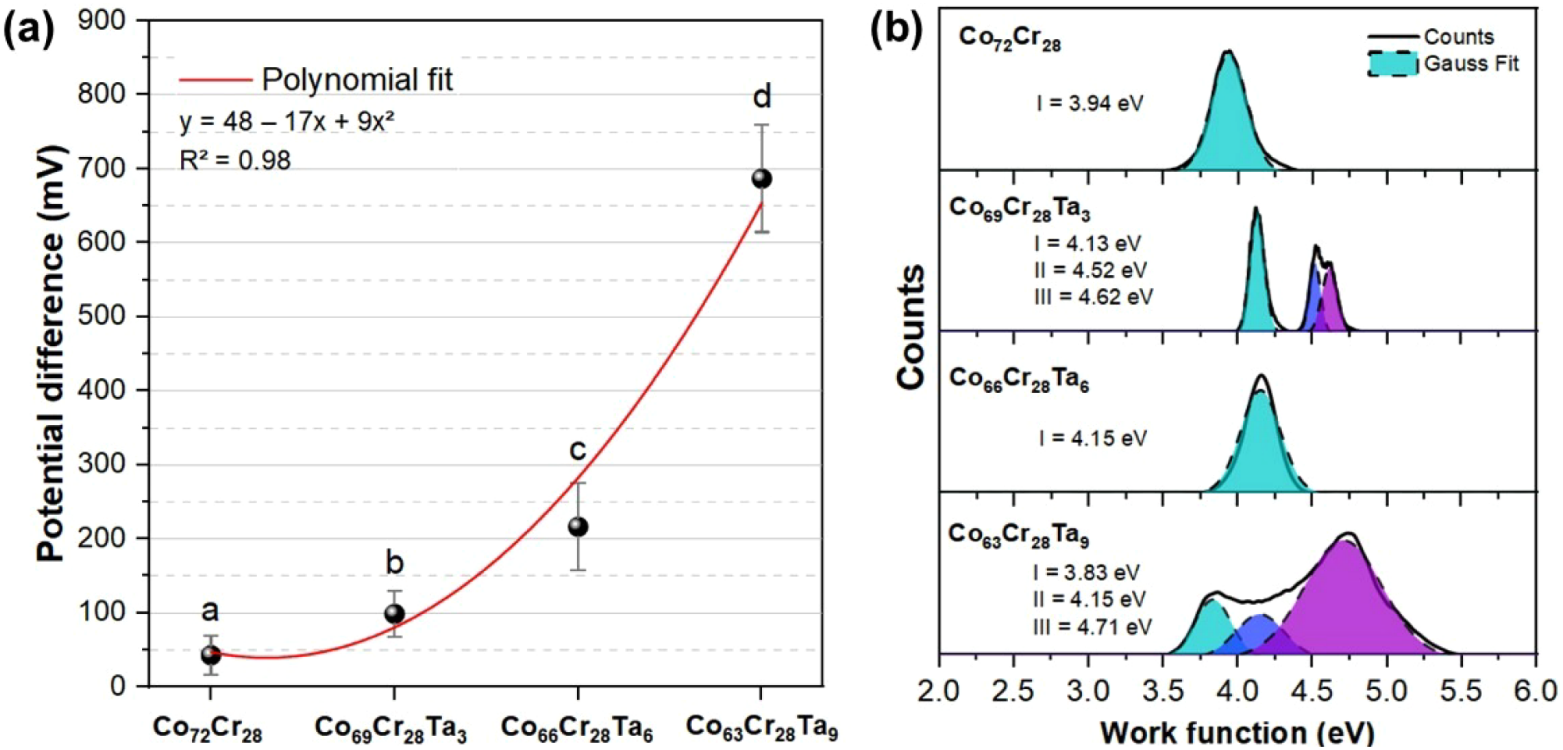
(a) Potential difference between the TaCo_2_ phase and Co–Cr matrix. The spheres indicate the mean value, the red line indicates the polynomial fit, while the bars are the standard deviations for the samples. Means followed by the same lowercase letter do not differ from each other by the Tukey test (*p* < 0.05). (b) Distribution of the work function obtained from potential maps for the samples, fitted with Gaussian functions.

To understand the potential values in addition to the standard line profile approach (Figures 3i–l, 4a), the work function distribution for 30 maps of each sample was obtained (Figure 4b), obtaining information about the work function of the entire map rather than only from points extracted from the line profile. The increase in the relative potential difference between the Co–Cr matrix and the TaCo_2_ phase (Figure 4a) is consistent with the increase in the average value of the work function distributions obtained from the complete maps (Figure 4b). Table S1 of the Supporting Information presents the descriptive parameters of the Gaussian fitting curves shown in Figure 4b. It is important to emphasize that the multiple Gaussian components observed for the Co_69_Cr_28_Ta_3_ and Co_63_Cr_28_Ta_9_ alloys do not necessarily correspond to distinct crystallographic phases, but rather to different populations of local work function values associated with microstructural heterogeneity, interfacial regions, and local compositional/electronic variations in the KPFM maps.

To avoid ambiguity, it is important to distinguish between the local contact potential difference (ΔP) and the uniformity of the work function. ΔP values were obtained from line profiles and represent the local electronic contrast between selected regions of the Co–Cr-rich matrix and regions of the TaCo_2_-rich precipitate. In contrast, the uniformity of the work function refers to the statistical distribution of work function values across the entire KPFM map, assessed from the Gaussian fit of pixel-by-pixel work function histograms. Therefore, a larger ΔP does not necessarily imply a less uniform distribution of the work function. The Co_66_Cr_28_Ta_6_ alloy exhibited a larger local ΔP than Co_69_Cr_28_Ta_3_, but its work function histogram was described by a single Gaussian population centered at 4.15 eV, with fwhm = 0.32 eV. This indicates a statistically continuous distribution of the work function over the mapped area. In contrast, the Co_69_Cr_28_Ta_3_ alloy required three Gaussian components centered at 4.13, 4.52, and 4.62 eV, with a total separation between peak centers of approximately 0.49 eV, indicating distinct electronic subpopulations. The Co_63_Cr_28_Ta_9_ alloy showed the greatest heterogeneity, with three Gaussian components centered at 3.83, 4.15, and 4.71 eV and a total separation between peak centers of approximately 0.88 eV (Table S1 of the Supporting Information). Therefore, in the present work, electronic heterogeneity was interpreted primarily from the multimodality of the histogram and peak separation, while ΔP was used to quantify local electronic contrast. This distinction explains why Co_66_Cr_28_Ta_6_ can exhibit measurable local potential differences, while also presenting a more spatially uniform work function distribution than Co_69_Cr_28_Ta_3_ and Co_63_Cr_28_Ta_9._

### 3.3 Wettability Assessment by Contact Angle Measurements

Figure 5 shows the contact angle values measured on the polished samples. It can be observed that the contact angle decreases as Ta is inserted into the Co–Cr matrix, with mean values and standard deviations of 76.18 ± 0.89 (Co_72_Cr_28_), 74.64 ± 0.95 (Co_69_Cr_28_Ta_3_), 73.39 ± 1.03 (Co_66_Cr_28_Ta_6_), and 70.88 ± 1.04° (Co_63_Cr_28_Ta_9_). The behavior of this property follows a polynomial function (y = 76 – 0.35x – 0.03x^2^, R^2^ = 0.98) for contact angle as a function of Ta concentration. It is observed that the polished samples exhibit hydrophilic characteristics (<90°) with increased wettability as a function of the Ta concentration.

**Figure 5:**
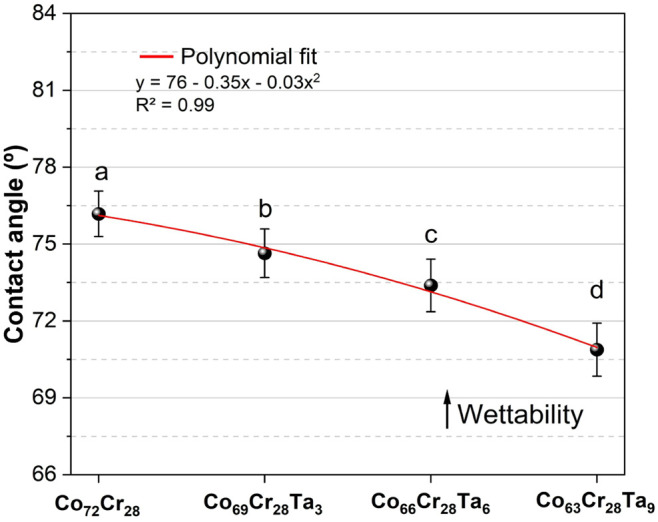
Droplet contact angle on the surface of polished samples: spheres indicate the mean value, while bars indicate the standard deviations for Co_72_Cr_28_, Co_69_Cr_28_Ta_3_, Co_66_Cr_28_Ta_6_, and Co_63_Cr_28_Ta_9_ samples. Means followed by the same lowercase letter do not differ from each other by Tukey’s test (*p* < 0.05).

### 3.4 Calcium Phosphate Deposition after SBF Immersion

The surface of the series of samples after immersion in SBF, as characterized by XRD (Figure 6a), shows the formation of calcium phosphate and sodium chloride (indicated by asterisks), and by SEM (Figure 6b) demonstrates the formation of globular aggregates, whereas in Co_63_Cr_28_Ta_9_, high contrast structures arise with irregular distributions. The aggregates decrease as the Ta concentration in the samples increases, resulting in the formation of high contrast structures in the sample with the highest Ta concentration (Co_63_Cr_28_Ta_9_). The EDS measurements (Figure 7a–d) demonstrate that the aggregates have the main elements phosphorus (P) and calcium (Ca), whereas the high contrast structures (Figure 7e) have the elements sodium (Na) and chlorine (Cl). In addition to these structures, it is seen that there is a distribution of the elements Ca and P as a film deposited on the surface (points 1, 3, 5, and 7 in Figure 7).

**Figure 6:**
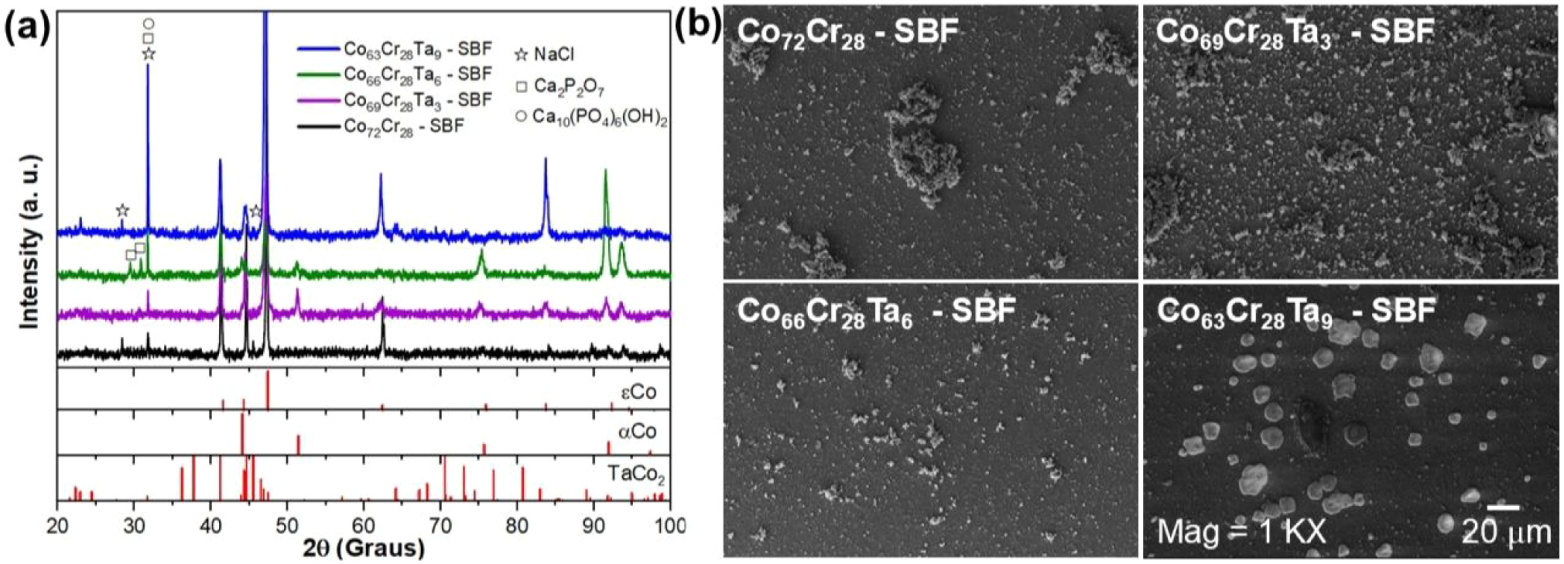
(a) X-ray diffraction patterns of the samples after immersion in SBF: the bar graphs represent the crystallographic phases εCo, αCo, and TaCo_2_ present in the Co_72_Cr_28_-, Co_69_Cr_28_Ta_3_-, Co_66_Cr_28_Ta_6_-, and Co_63_Cr_28_Ta_9_–SBF samples. (b) Scanning electron micrographs (magnification 1000x) of the samples after immersion in SBF: Co_72_Cr_28_–SBF, Co_69_Cr_28_Ta_3_–SBF, Co_66_Cr_28_Ta_6_–SBF, and Co_63_Cr_28_Ta_9_–SBF.

**Figure 7:**
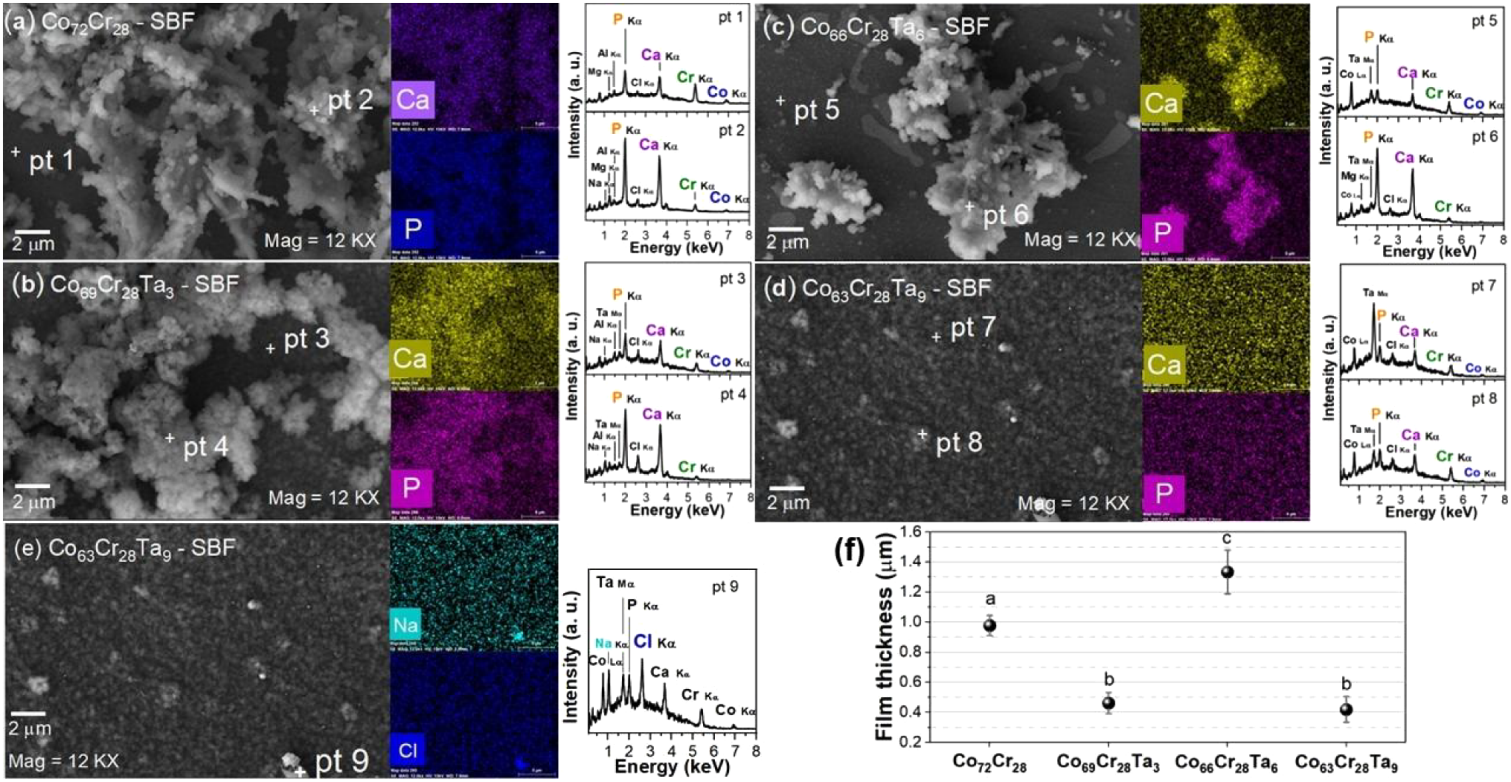
Morphological and elemental information on the surface (magnification of 12000×) of the samples after immersion in SBF: surface microstructure image, compositional maps, and points 1–8 described by EDS of (a) Co_72_Cr_28_-, (b) Co_69_Cr_28_Ta_3_-, (c) Co_66_Cr_28_Ta_6_-, and (d–e) Co_63_Cr_28_Ta_9_–SBF. (f) Thicknesses of the films deposited on the surfaces of the samples after immersion in SBF for 28 days: The spheres indicate the mean value, and the bars are the standard deviations for the samples. Means followed by the same lowercase letter do not differ from each other by Tukey’s test (*p* < 0.05).

From the EDS spectra, it was possible to extract the Ca/P ratio of the calcium phosphate nucleated on the surface, highlighting that the equipment was not calibrated for the chemical elements, taking into account here the relative intensities of Ca and P. The calculated Ca/P ratio values were 1.84 (Co_72_Cr_28_–SBF), 1.84 (Co_69_Cr_28_Ta_3_–SBF), 1.83 (Co_66_Cr_28_Ta_6_–SBF), and 1.85 (Co_63_Cr_28_Ta_9_–SBF). Furthermore, using the AFM technique, it was possible to measure the film thicknesses (Figure 7f) as 0.98 ± 0.07 μm (Co_72_Cr_28_), 0.46 ± 0.07 μm (Co_69_Cr_28_Ta_3_), 1.33 ± 0.15 μm (Co_66_Cr_28_Ta_6_), and 0.42 ± 0.09 μm (Co_63_Cr_28_Ta_9_). The films presented thicknesses in the micrometric scale, thicker in Co_72_Cr_28_ and Co_66_Cr_28_Ta_6_.

### 3.5 Fibroblast Cell Viability under Alloy Extract Exposure

Figure 8 shows cell survival after 24 and 48 h of interaction with sample extracts. After 24 h, the Co_72_Cr_28_, Co_69_Cr_28_Ta_3_, and Co_66_Cr_28_Ta_6_ samples increased cell viability by more than 125%; however, the Co_63_Cr_28_Ta_9_ sample remained unchanged from the control (98%). Over 48 h, a trend of increased cell viability directly proportional to Ta content was found, with a peak of 165% in the Co_63_Cr_28_Ta_9_ sample. At both analysis intervals, all samples had cell viability equivalent to or greater than the negative control, indicating that the alloys are biocompatible with the fibroblast cell line. A statistically significant difference was observed between the Co_72_Cr_28_ alloy and the Ta-containing samples, with the Co_66_Cr_28_Ta_6_ sample showing intermediate behavior, with no significant difference for either group. As expected, all samples differed significantly from the positive control. The CoCr alloy demonstrated biocompatibility equivalent to the negative control (pure medium), while the Co_69_Cr_28_Ta_3_ and Co_63_Cr_28_Ta_9_ samples also showed highly biocompatible results, with no significant difference between them.

**Figure 8:**
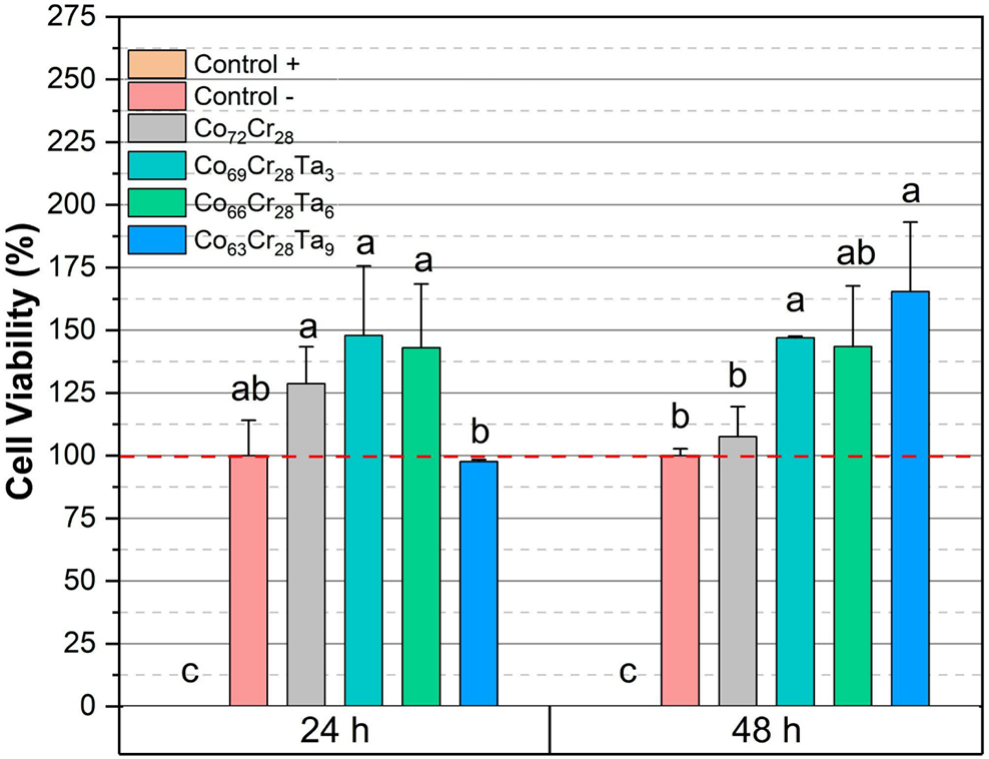
Cell survival tests after 24 and 48 h of cell culture in the samples: The columns indicate the mean value, and the bars are the standard deviations for the samples Co_72_Cr_28_, Co_69_Cr_28_Ta_3_, Co_66_Cr_28_Ta_6_, and Co_63_Cr_28_Ta_9_. The dotted line signals cell survival for the negative control. Means followed by the same lowercase letter do not differ from each other by Tukey’s test (*p* < 0.05).

## 4 Discussion

The XRD patterns and phase diagrams of Co–Cr and Co–Ta systems^31^ indicate that Cr dissolves in the Co lattice, whereas Ta, being above the solubility limit of Co allotropes, precipitates as the intermetallic phase TaCo_2_ (Figure 1a). The simultaneous presence of εCo and αCo suggests that cooling was not fast enough to suppress the allotropic transformation, yielding a dual-phase microstructure. TaCo_2_ corresponds to a Laves phase (AB_2_ stoichiometry), consistent with the atomic size ratio of 1.1. Previous studies on Co–Cr–W alloys also showed that Ta contents above 4 wt % trigger Laves precipitation,^4^ in agreement with our observations.

Scanning electron microscopy revealed dendritic segregation, with Ta-rich regions corresponding to TaCo_2_, while interdendritic regions consisted of Co–Cr matrix phases (εCo and αCo) (Figure 2). Although XRD detected TaCo_2_ only from 6 wt % Ta, SEM/EDS revealed its presence already at 3 wt % Ta, likely below the detection limit of XRD (∼2 vol %).^32^ Quantitative SEM image analysis estimated an apparent TaCo_2_ area fraction of 1.26 ± 0.40% for Co_69_Cr_28_Ta_3_. Considering standard stereological principles, this area fraction may be used as an approximate estimate of the volume fraction in representative polished sections, corresponding to approximately 1.3 vol % TaCo_2_. This value is close to or below the practical detection limit of laboratory XRD for a minor phase in a bulk multiphase alloy. In contrast, the Co_66_Cr_28_Ta_6_ alloy exhibited a higher apparent TaCo_2_ fraction, 3.70 ± 0.37%, for which TaCo_2_ diffraction peaks became detectable. Therefore, under the present experimental conditions, the practical XRD detection threshold for TaCo_2_ lies between approximately 1.3 and 3.7 vol %. The absence of TaCo_2_ peaks in Co_69_Cr_28_Ta_3_ does not indicate the absence of this phase, but rather its low fraction and limited diffracted intensity. This result reinforces the complementary nature of XRD and SEM/EDS: XRD identifies crystallographic phases above the detection limit, while SEM/EDS reveals local chemical segregation and minor phase precipitation.

The observed segregation is attributed to both the solubility limit of Cr and Ta in Co and to the differences in melting points (Co: 1495 °C, Cr: 1907 °C, Ta: 3020 °C), where high-melting Ta crystallizes during the early solidification stages.^33,34^ Furthermore, increasing Ta content promotes a progressively more interconnected spatial distribution of TaCo_2_ precipitates, increasing the density of intermetallic interfaces (Figure S5, Supporting Information). Such dendritic structures are typical of biomedical Co–Cr alloys produced by casting, such as ASTM F75.^35^

The microhardness values obtained (up to ∼466 HV at 9 wt % Ta) (Figure 1b) are significantly higher than those of conventional biomedical Co–Cr alloys, such as ASTM F75 and F90 (300–400 HV).^36^ The high hardness values, as well as the nonlinear increase in hardness with Ta addition, can be qualitatively attributed to coupled structural factors, including the progressive formation of the TaCo_2_ intermetallic phase, possible variations in the relative fraction of Co allotropes, and grain-size effects.^37,38^ Among these factors, TaCo_2_ precipitation is the most directly supported by the present SEM/EDS and image-analysis results, which show an increasing fraction and spatial connectivity of Ta-rich intermetallic regions with increasing Ta concentration. This phase likely contributes to hardness enhancement through precipitation strengthening and by acting as an obstacle to dislocation motion, as commonly reported for Laves-type intermetallic phases.^39,40^

The larger absolute standard deviation observed for the Co_63_Cr_28_Ta_9_ alloy is consistent with its more heterogeneous microstructure and higher fraction of TaCo_2_-rich regions. Because Vickers microhardness is a localized indentation measurement, each indentation may probe different local proportions of Co–Cr matrix, TaCo_2_-rich precipitates, and phase boundaries. Nevertheless, this variability does not compromise the hardness trend, since the relative dispersion remained comparable among the samples and the differences in mean hardness were statistically significant by Tukey’s test. Thus, TaCo_2_-related microstructural heterogeneity contributes to local mechanical variability, but the overall increase in hardness with Ta content remains statistically robust. In biomedical contexts, higher hardness is relevant to wear resistance in articulating environments, improving implant lifetime.

KPFM mapping revealed a pronounced increase in potential differences with Ta doping, reaching ∼687 mV at 9 wt % Ta, a ∼1500% increase compared to the base alloy (Figures 3, 4a). The nonlinear increase in contact potential difference reflects the coupled evolution of microstructural heterogeneity, observed by SEM/EDS, and electronic heterogeneity, detected by KPFM. Ta addition promotes the coexistence of Co–Cr-rich matrix regions and TaCo_2_-rich precipitates, which possess distinct local electronic structures and therefore generate local differences in surface potential. Possible changes in the relative fraction of Co allotropes may also contribute to local electronic variations. In addition to the increase in TaCo_2_ fraction, the progressive change in the spatial distribution and connectivity of TaCo_2_ precipitates may amplify the electronic contrast detected by KPFM. Thus, the nonlinear evolution of contact potential difference is interpreted as a consequence of the coupled chemical, structural, and morphological heterogeneity induced by Ta addition, rather than as the result of a single isolated factor. Consistently, a quadratic dependence on Ta concentration was also observed for the TaCo_2_ phase fraction estimated by ImageJ analysis, as well as for the Vickers microhardness, contact potential difference, and contact angle data (Figure S6 and Table S2 of the Supporting Information).

The TaCo_2_ phase fraction, Vickers microhardness, and contact potential difference all increased with Ta addition, but with markedly different rates. While microhardness increased from 263 ± 20 HV0.5 for Co_72_Cr_28_ to 466 ± 27 HV0.5 for Co_63_Cr_28_Ta_9_, corresponding to an increase of approximately 77%, the contact potential difference increased from 43 ± 27 to 687 ± 73 mV, corresponding to an increase of approximately 1500%. This difference indicates that the mechanical and electronic responses are governed by related but distinct mechanisms. Vickers microhardness is a volume-averaged mechanical property,^41^ reflecting the collective resistance of the Co–Cr matrix and TaCo_2_-rich precipitates to plastic deformation. In this case, TaCo_2_ contributes to strengthening by acting as a hard intermetallic phase and by obstructing dislocation motion, but the measured hardness remains moderated by the surrounding matrix, precipitate spacing, and phase-boundary interactions.^37,38^ In contrast, the contact potential difference measured by KPFM is a local surface-sensitive electronic response.^16^ It is strongly affected by the electronic contrast between Co–Cr matrix regions and TaCo_2_ precipitates, as well as by the density and connectivity of matrix/intermetallic interfaces. Therefore, small changes in TaCo_2_ distribution and connectivity can amplify local electrostatic contrast much more strongly than they affect the averaged mechanical response. This explains why TaCo_2_ precipitation produces a moderate nonlinear increase in hardness but a much steeper nonlinear increase in contact potential difference.

Work function mapping provided further evidence of the role of Ta in modulating surface electronic properties. The distribution of Φ values (Figure 4b) was very sensitive to microstructural features such as crystallographic phases and TaCo_2_ precipitates, generating heterogeneous electronic landscapes. Interestingly, although TaCo_2_ precipitation is clearly observed in the Co_66_Cr_28_Ta_6_ alloy, this sample exhibits a homogeneous work-function distribution. This behavior suggests that the KPFM response is governed not only by the presence of TaCo_2_ precipitates but also by their size, spatial distribution, connectivity, and electronic coupling with the Co–Cr matrix. The Co_66_Cr_28_Ta_6_ alloy appears to represent an intermediate microstructural regime in which TaCo_2_-rich regions are sufficiently present to modify the surface electronic landscape but remain finely and relatively uniformly distributed throughout the matrix. Under these conditions, the local electronic contrast between the Co–Cr-rich matrix and TaCo_2_-rich precipitates may be spatially averaged within the capacitance-weighted KPFM signal, resulting in a single dominant work-function population. In contrast, the Co_69_Cr_28_Ta_3_ alloy contains fewer and more irregularly distributed Ta-rich regions, favoring discrete electronic subpopulations, whereas the Co_63_Cr_28_Ta_9_ alloy exhibits a higher fraction and greater connectivity of TaCo_2_ precipitates (Figure S5 of the Supporting Information), which amplifies the local electronic contrast and leads to a more heterogeneous work-function distribution. Therefore, the homogeneous work-function distribution observed for Co_66_Cr_28_Ta_6_ is attributed to a balance between TaCo_2_ precipitation, uniform precipitate dispersion, interfacial electronic equilibration, and KPFM spatial averaging, rather than to the absence of microstructural heterogeneity.

The local work-function distribution should not be attributed exclusively to TaCo_2_ precipitation. Other structural factors, including the coexistence of εCo and αCo allotropes, crystallographic orientation, grain structure, and passive oxide composition, may also contribute to local electronic variations.^26,42^ However, a quantitative separation of the individual contributions of Co allotrope ratio and TaCo_2_ precipitation would require additional phase- and orientation-resolved analyses, such as Rietveld refinement, Electron Backscatter Diffraction (EBSD), and correlative KPFM/EBSD mapping. Within the present data set, TaCo_2_ precipitation remains the most directly supported contributor to the observed electronic heterogeneity because the strongest potential contrasts are spatially associated with Ta-rich intermetallic regions identified by SEM/EDS. In contrast, the Co_72_Cr_28_ alloy, despite the coexistence of Co allotropes, exhibited a comparatively homogeneous work-function distribution and the lowest potential difference. Therefore, although Co allotropes may contribute to the baseline electronic response of the Co–Cr matrix, the pronounced increase in local electronic heterogeneity with Ta addition is mainly associated with the emergence, spatial distribution, and connectivity of TaCo_2_-rich regions (Figure S5 of the Supporting Information).

Interestingly, the Ca/P-rich layer thickness did not follow the same monotonic trend observed for wettability. The contact angle decreased from 76.18 ± 0.89° for Co_72_Cr_28_ to 70.88 ± 1.04° for Co_63_Cr_28_Ta_9_, indicating an approximately 7% increase in wettability across the alloy series. However, the thickest Ca/P-rich layer was not observed for the most hydrophilic sample. Instead, Co_66_Cr_28_Ta_6_, with an intermediate contact angle of 73.39 ± 1.03°, formed the thickest Ca/P-rich layer, 1.33 ± 0.15 μm, whereas Co_63_Cr_28_Ta_9_ formed a much thinner layer, 0.42 ± 0.09 μm. In addition, the samples with more homogeneous work-function distributions, Co_72_Cr_28_ and Co_66_Cr_28_Ta_6_, formed thicker Ca/P-rich layers, 0.98 ± 0.07 μm and 1.33 ± 0.15 μm, respectively, while the samples with more heterogeneous work-function distributions, Co_69_Cr_28_Ta_3_ and Co_63_Cr_28_Ta_9_, formed thinner layers, 0.46 ± 0.07 μm and 0.42 ± 0.09 μm, respectively. Therefore, the average Ca/P-rich layer thickness of the electronically more homogeneous samples was approximately 2.6 times higher than that of the electronically heterogeneous samples.

Although contact angle, absolute work function, and work-function distribution are related surface descriptors, their independent contributions to wettability cannot be quantitatively separated from the present data set because these parameters evolve simultaneously with Ta concentration and TaCo_2_ precipitation. Moreover, only the water contact angle was measured; therefore, a full decomposition of surface energy into polar and dispersive components would require additional liquids and a multicomponent surface-energy model. Nevertheless, using the Young-Dupré relationship as a surface-energy-related indicator,^43^ the apparent work of adhesion of water increased from approximately 90.2 to 96.6 mJ m^–2^ across the same compositional range. This increase is consistent with Ta-related changes in the average surface state. However, because the outermost surface chemistry, hydroxylation, and passive oxide composition or thickness were not directly measured, the specific physicochemical origin of the wettability change cannot be established from the present data set.

These results indicate that wettability may contribute to Ca/P nucleation but is unlikely to be the only factor associated with layer growth. Hydrophilic surfaces are generally associated with improved ion adsorption and calcium-phosphate nucleation.^44^ However, Ca/P formation may also be influenced by local surface chemistry, topography, and electrostatic/electronic heterogeneity.^45,46^ Because all samples were polished under identical conditions, the observed differences are consistent with possible Ta-associated changes in surface state and electronic response rather than with macroscopic topographic differences. A heterogeneous work-function distribution may generate local electrostatic microdomains with different affinities for Ca^2+^ and phosphate species, potentially favoring discontinuous nucleation and isolated deposits instead of a spatially continuous Ca/P-rich layer. However, direct confirmation of this interpretation would require complementary measurements of passive-layer chemistry, ion adsorption, and time-resolved nucleation behavior.

In contrast, work-function distribution reflects local nanoscale electronic heterogeneity. Therefore, the fact that Co_63_Cr_28_Ta_9_ exhibits both the highest hydrophilicity and the strongest work-function heterogeneity does not represent a contradiction. Contact angle is a macroscopic, averaged response, whereas KPFM detects local electronic contrast between microstructural regions. Thus, stronger electronic heterogeneity may coexist with higher hydrophilicity while still hindering the formation of a spatially continuous Ca/P-rich layer due to nonuniform ion adsorption sites. Thus, the present trends are consistent with the possibility that macroscopic wettability and nanoscale work-function distribution contribute differently to Ca/P-rich layer formation. Under the conditions investigated, Co_66_Cr_28_Ta_6_ showed the most favorable combination of increased wettability, a more continuous work-function distribution, and greater Ca/P-rich layer thickness. This association does not establish the independent mechanistic contribution of each surface parameter, which will require further direct investigation.

Regarding this data, the average Ca/P ratio (∼1.8) indicates mixed calcium phosphate phases, possibly including Ca_2_P_2_O_7_ (Figure 6a), a nontoxic bioactive compound considered a precursor of hydroxyapatite.^47–49^ Although the apparent Ca/P ratios obtained from EDS were very similar among the samples, ranging from approximately 1.830 to 1.850, the Ca/P-rich layer thickness varied markedly, from 0.42 ± 0.09 μm for Co_63_Cr_28_Ta_9_ to 1.33 ± 0.15 μm for Co_66_Cr_28_Ta_6_. It is important to note that these Ca/P ratios should be interpreted as semiquantitative values, since the EDS analysis was not calibrated for absolute elemental quantification. Within this limitation, the results indicate that Ta addition and the associated work-function distribution are correlated with variations in the final thickness and spatial continuity of the Ca/P-rich layer. These observations may reflect differences in early nucleation density and growth behavior, but the precipitation kinetics were not directly measured. This behavior is consistent with the fact that the final composition of calcium-phosphate deposits is strongly influenced by the ionic composition, pH, supersaturation state, and immersion time in SBF,^22^ whereas the alloy surface primarily controls the early interfacial events that determine where and how the Ca/P layer nucleates and grows.^46^ Therefore, Ta-induced surface electronic properties appear to regulate Ca/P growth kinetics and layer continuity more strongly than the final apparent Ca/P ratio.

It is important to emphasize that the SBF assay provides an *in vitro* indication of a surface’s ability to induce calcium phosphate deposition but does not directly prove superior *in vivo* osseointegration. Therefore, the thicker Ca/P-rich layer observed for Co_66_Cr_28_Ta_6_, 1.33 ± 0.15 μm, should be interpreted as evidence of greater *in vitro* Ca/P deposition capacity under the SBF conditions used, and not as direct proof of better bone integration. Further osteogenic assays and *in vivo* studies would be necessary to confirm whether this response translates into enhanced osseointegration. Furthermore, all SBF experiments were performed under identical solution composition, temperature, immersion time, and sample/solution ratio, and pH was monitored during immersion. Therefore, differences in the thickness of the Ca/P-rich layer are more consistently associated with intrinsic alloy surface properties, such as wettability and electronic heterogeneity, than with differences in bulk SBF conditions. However, since the kinetics of precipitation resolved as a function of pH was not the focus of this study, a possible contribution from local pH variations at the alloy/electrolyte interface cannot be completely ruled out.

Cell viability assays confirmed the cytocompatibility of all samples, with viability values close to or higher than the negative control (Figure 8). According to the literature,^25^ materials are considered cytotoxic only when viability falls below 70%. However, the cellular response was time-dependent. After 24 h, Co_72_Cr_28_, Co_69_Cr_28_Ta_3_, and Co_66_Cr_28_Ta_6_ showed an increase in the MTT signal, while Co_63_Cr_28_Ta_9_ remained close to the negative control. After 48 h, cell viability increased with Ta content, reaching the highest value for Co_63_Cr_28_Ta_9_. This behavior suggests that surface potential heterogeneity does not act as a linearly positive parameter but rather modulates the cellular response in a time-dependent manner.

Based on the KPFM results presented, low to moderate potential differences, up to approximately 217 ± 59 mV, were consistent with an enhanced initial response to the MTT assay, while the much larger potential difference observed for Co_63_Cr_28_Ta_9_, 687 ± 73 mV, did not intensify the response after 24 h. Thus, the results suggest the existence of an ideal empirical range of surface potential heterogeneity for the initial cellular response in this alloy system. However, this range should not be interpreted as a universal threshold, since only four alloy compositions and two exposure times were evaluated.

Since the biological assays were performed using alloy extracts instead of direct cell-surface contact, the observed response cannot be directly attributed solely to the local heterogeneity of the surface potential. Instead, the results indicate an indirect association between the electronic characteristics measured on the alloy surfaces and the extract-mediated biological response. This association may reflect physicochemical processes occurring during extract formation, including possible differences in alloy-medium interfacial reactivity, passive-film behavior, and ion release.^50,51^ However, these processes were not directly measured, and neither the chemical composition of the extracts nor the specific surface features responsible for the observed MTT response can be determined from the present data set. In this context, the heterogeneous distribution of the work function observed for the samples containing Ta may influence the physicochemical processes occurring at the alloy/electrolyte interface, consequently affecting the extract composition and cellular metabolic activity.

Previous studies have shown that heterogeneous surface potential distributions can mimic aspects of the natural extracellular microenvironment and promote protein adsorption and cellular activity.^52,53^ In particular, Bai et al.^53^ demonstrated that electrically heterogeneous surfaces can increase protein adsorption and stimulate cell spreading behavior. Although current biological assays have been performed using alloy extracts instead of direct cell-surface contact, these previous findings corroborate the hypothesis that the electronic heterogeneity of the alloy surface can indirectly influence the biological response through physicochemical processes that occur at the alloy/electrolyte interface during extract formation.

Therefore, the current results do not contradict the hypothesis that electronic surface heterogeneity influences the biological response but indicate that this effect is associated with other physicochemical factors and evolves with exposure time. Future studies involving direct contact assays, quantification of ion release, protein adsorption, focal adhesion markers, cytoskeleton staining, and long-term biological assessments will be necessary to elucidate the relationship between surface electronic heterogeneity and fibroblast behavior in this alloy system.

## 5 Conclusion

This study demonstrates that Ta addition is an effective strategy for tailoring the microstructure, surface electronic properties, *in vitro* bioactivity, and cytocompatibility of Co–Cr alloys. The incorporation of Ta promoted the formation of a TaCo_2_ intermetallic phase and significant microstructural changes, resulting in a 77% increase in Vickers microhardness, which indicates enhanced resistance to localized plastic deformation. KPFM analysis showed that Ta addition also modified the surface electronic landscape, producing composition-dependent changes in contact potential difference and work-function distribution.

Immersion in simulated body fluid showed that all alloys promoted calcium-phosphate deposition, with Co_66_Cr_28_Ta_6_ presenting the thickest Ca/P-rich layer, 1.33 ± 0.15 μm. This behavior was associated with a favorable combination of wettability and work-function distribution, suggesting that these surface descriptors may contribute to Ca/P-rich layer formation under the present SBF conditions. Cell viability assays confirmed the cytocompatibility of the alloys under the conditions tested, and the Ta-containing compositions suggested that surface electronic heterogeneity may modulate cell response in a time-dependent manner.

Although the hardness results indicate improved resistance to localized deformation, they do not by themselves demonstrate full mechanical suitability for load-bearing biomedical implants. Additional mechanical characterization, including elastic modulus, strength, ductility, fracture/damage tolerance, and fatigue resistance, remains necessary to determine the performance of these alloys under clinically relevant loading conditions. Overall, among the investigated compositions, Co_66_Cr_28_Ta_6_ exhibited the most balanced combination of microhardness, *in vitro* Ca/P deposition, work-function distribution, and cytocompatibility, highlighting Co–Cr–Ta alloys as promising candidates for future biomedical investigations.

## Supplementary Material



## Notes

KPFM potential-to-work-function conversion and calibration using the Al–Si–Au reference grid (Figures S1–S4); Gaussian fitting parameters for KPFM-derived work-function distributions (Table S1); SEM micrographs showing the spatial distribution of the Co–Cr matrix and TaCo_2_ precipitates (Figure S5); polynomial relationships among apparent TaCo_2_ area fraction, Vickers microhardness, contact potential difference, and contact angle (Figure S6 and Table S2) (PDF)
