# Supplementary material for: Tantalum-Doped Co–Cr Alloys: Multiscale Effects on Mechanics, Surface Potential, and Biological Responses
Source: ACS Omega. 2026 Jul 15;11(29):43598–610. doi: 10.1021/acsomega.6c02438 (PMC13425330; doi:10.1021/acsomega.6c02438)
Supplement: Supplementary file 1 [file ao6c02438_si_001.pdf]

Tantalum-Doped Co-Cr Alloys: Multiscale Effects on Mechanics, Surface Potential, and Biological Responses

Beatriz da Silva Batista[a,b*], Rosa Maria Viana Sousa[b], Samuel Filgueiras Rodrigues[c], Napoleão Martins Argôlo Neto[d,e,f], Alan Silva de Menezes[a,b], Luzeli Moreira da Silva[a], Pierre Basílio Almeida Fechine[g], Ralph Santos-Oliveira[h,i], Luciana Magalhães Rebêlo Alencar[a,b**]

[a] Center for Social Sciences, Health and Technology, Federal University of Maranhão, Advanced Unit, Imperatriz 65915-060, MA, Brazil;
[b] Department of Physics, Laboratory of Biophysics and Nanosystems, Federal University of Maranhão, Campus Bacanga, São Luís 65085-580, MA, Brazil;
[c] Federal Institute of Education, Science and Technology of Maranhão, Monte Castelo, São Luís 65030-005, MA, Brazil;
[d] Postgraduate Program in Technologies Applied to Animals of Regional Interest, Federal University of Piauí, Teresina 64049-550, PI, Brazil.
[e] Federal University of Piauí, Bachelor of Veterinary Medicine, Teresina 64049-550, PI, Brazil.
[f] Integrated Center for Morphology and Stem Cell Research, Federal University of Piauí, Teresina 64049-550, PI, Brazil
[g] Advanced Materials Chemistry Group, Department of Analytical Chemistry and Physical Chemistry, Federal University of Ceará, Campus do Pici, Fortaleza 60455-760, CE, Brazil
[h] Brazilian Nuclear Energy Commission, Institute of Nuclear Engineering, Laboratory of Nanoradiopharmacy and Synthesis of New Radiopharmaceuticals, Rio de Janeiro, Brazil;
[i] State University of Rio de Janeiro, Laboratory of Radiopharmacy and Nanoradiopharmaceuticals, Rio de Janeiro, Brazil.

Corresponding authors: batistas.bea@gmail.com, luciana.alencar@ufma.br***.

SUPPLEMENTARY MATERIALS

1. Conversion of raw potential data into work function – KPFM

The work function values were obtained using the potential-to-work function conversion option ("Set input offset") available in the Nanoscope Analysis 2.0 software (Bruker) [1]. As this procedure involves the relationship between the measured surface potential and the work functions of the probe and the sample, as described by Equation 1, the conversion procedure used in this work is detailed below.

$$V_{CPD} = \frac{\Phi_1 - \Phi_2}{e}, \qquad \text{Equation 1}$$

where V_{CPD} is the contact potential difference between the sample and the tip, and is the elementary charge, Φ_1 and Φ_2 are the work functions of the sample and the tip, respectively.

During the characterization of the samples developed in this work, a protocol was sought to measure the work function of the samples from the KPFM measurement. As a validation step, the potential data (V_{CPD}) were converted into a work function for an aluminum-silicon-gold (Al-Si-Au) reference sample, measured in air. Figure S1 shows the (a) topography and (b) potential maps, as well as the (c-d) distribution of the corresponding data.

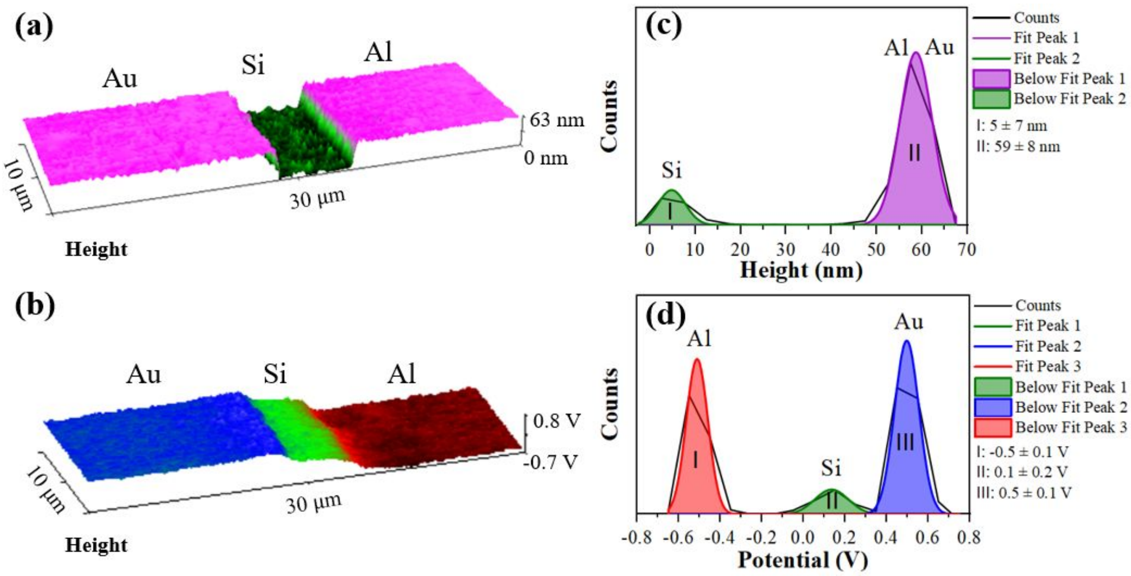

Figure S1. Al-Si-Au grid data obtained from the KPFM: maps of (a) topography and (b) potential, and histogram distributions of (c) height and (d) potential. The histograms were fitted using multiple Gaussian components.

The height histogram (Figure S1-c) shows the topographic difference of the grid, where Si occupies the lowest part, while Au and Al occupy the highest part. The potential histogram (Figure S1-d), in turn, reveals distinctions in the electrical properties of the three elements, evidenced by the adjustment of three Gaussians. The average V_{CPD} values obtained were: 0.5 ± 0.1 V (Au), 0.1 ± 0.2 V (Si), and -0.5 ± 0.1 V (Al), measured in relation to the tip. In this measurement configuration (PFQNE-Al tip, atmospheric environment, polarization applied to the tip), it is observed that the most positive potential is associated with Au.

According to Equation 1, the term V_{CPD} is the parameter quantified by KPFM during voltage adjustment in the measurement. Given that the elementary charge (e) and the tip work function (Φ_2) are known parameters, the only variable to be determined is the sample work function (Φ_1). To characterize the composition of the tip apex material

(the region of greatest interaction with the sample), EDS analysis was performed as shown in Figure S2, identifying the significant presence of Al in the cantilever, as well as in the tip apex (Figure S2-c). Therefore, the Al work function value was adopted for the conversion of the V_{CPD} data.

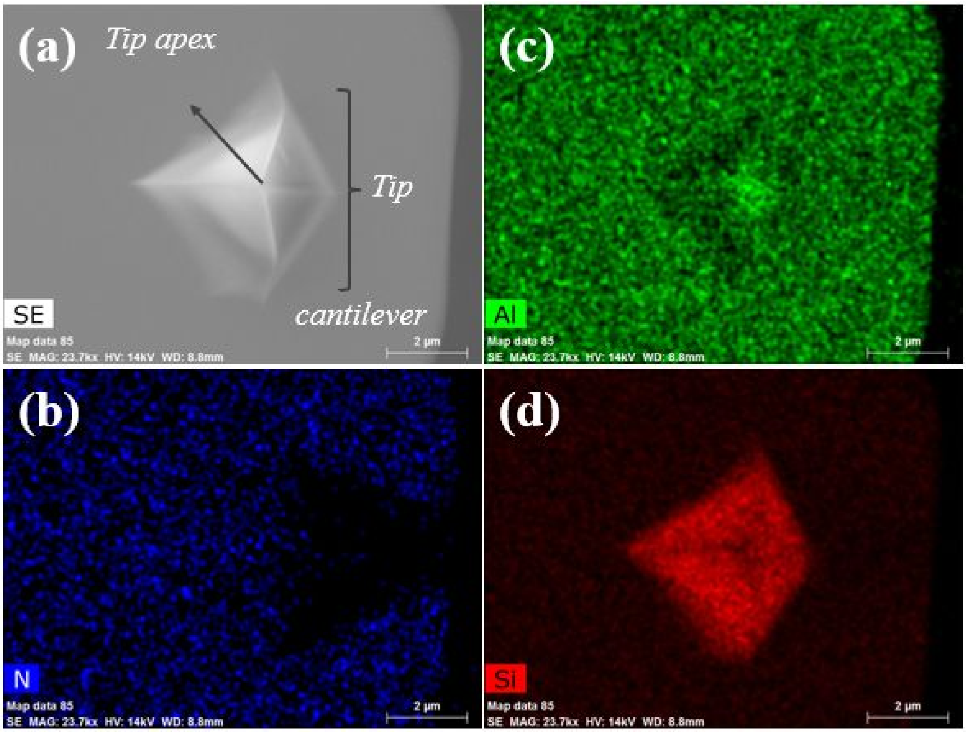

Figure S2. Compositional maps of the tip used in KPFM measurement by EDS: (a) secondary electrons (SE), (b) nitrogen (N), (c) aluminum (Al), and (d) silicon (Si).

The Nanoscope Analysis software offers the Set input offset tool, which allows you to enter the information of the probe used. Based on the previous chemical characterization of the probe (containing Al), the following steps were performed to convert the data: (1) the KPFM measurement of the Al-Si-Au standard grid was performed; (2) the reference value for Al (2.54 V) was entered in the Set input offset function; (3) the resulting potential map was saved; (4) the data was exported to a text document; (5) the data was processed in a graphical analysis software; and (6) the potential (V) values were converted to work function (eV) by multiplying by the elementary charge ($e = 1.6 \times 10^{-19}$ C).

The work function values (Figure S3) were obtained by fitting three Gaussian functions, revealing: 3.7 ± 0.3 eV (Al), 4.6 ± 3 eV (Si) and 5.4 ± 0.3 eV (Au). These

values agree with data reported in the literature [2]: 3.9 eV (Al), 4.6 eV (Si) and 5.2 eV (Au), validating the applied methodology.

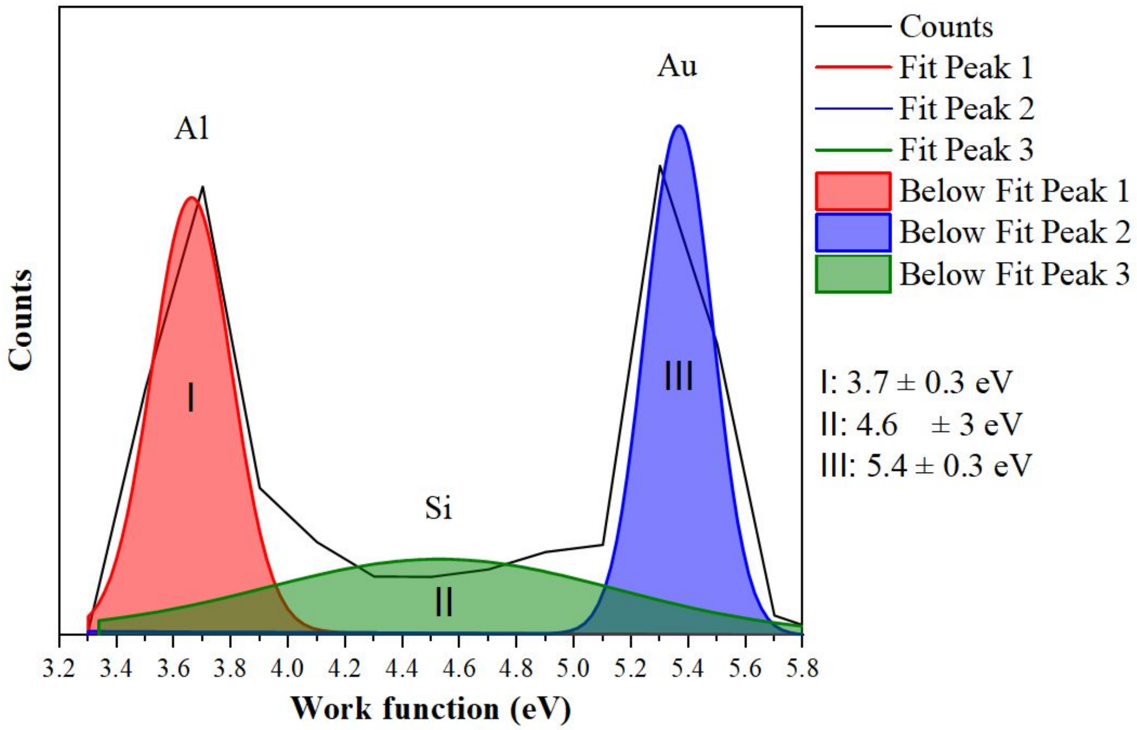

Figure S3. Histogram of the work function of the Al-Si-Au grid obtained from the KPFM measurement. The histogram was fitted using three Gaussian components to account for the different electronic populations associated with the Al, Si, and Au regions.

The same protocol was applied to the samples investigated in this work. Analyzing the extremes of the sample series, $Co_{72}Cr_{28}$ (3.94 ± 0.28 eV) and $Co_{63}Cr_{28}Ta_9$ (I: 3.83 ± 0.29 eV, II: 4.15 ± 0.38 eV and III: 4.71 ± 0.62 eV) (Figure S4), it is observed that the values are within the range reported for the pure elements, Co (4.52 eV and 4.85 eV), Cr (4.11 eV and 3.94 eV) and Ta (4.26 eV) [2]. However, the specific assignment of each adjustment peak (I, II, and III in Figure S4) to the individual elements is not straightforward, since the work function is a property influenced by several parameters, such as crystallographic orientation, presence of impurities or adsorbents, local chemical composition, and electronic states [2,3]. In the calibration grid (Al-Si-Au), direct assignment is possible (Figure S3) due to the well-defined distribution of the elements, while in alloys, there is the formation of a solid solution (Co-Cr matrix) and an intermetallic compound ($TaCo_2$) that introduces electronic modifications and influences the calculated work function.

Therefore, the importance of precise probe characterization, control of environmental conditions during measurement, and the use of a reference sample for calibration is highlighted. Furthermore, measuring the Au reference before and after KPFM scans is essential to assess instrumental stability, potential tip drift, and calibration uncertainty during acquisition, ensuring the reliability and reproducibility of the CPD/function data.

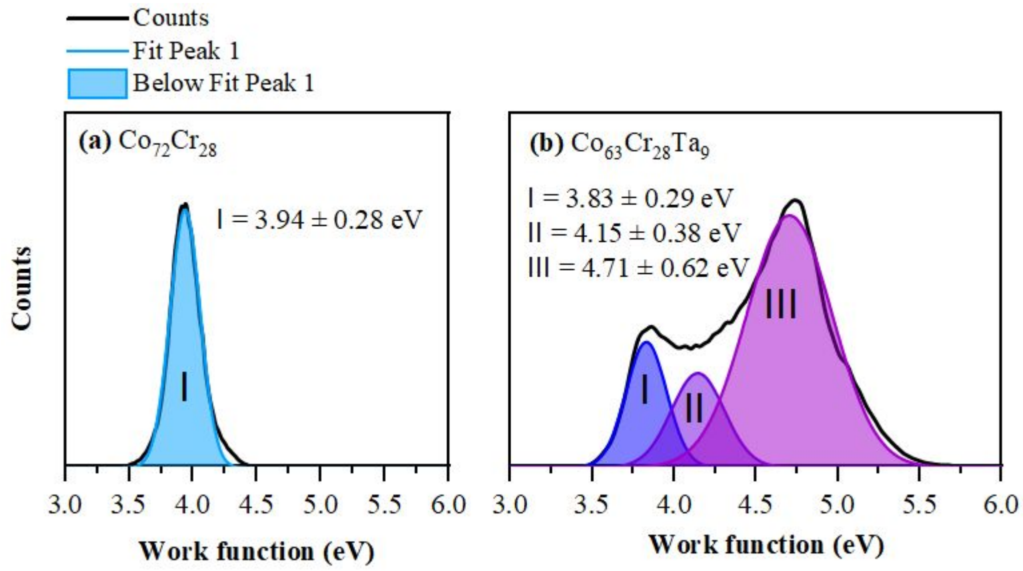

Figure S4. Histograms of the work function of the alloy samples obtained from the KPFM measurement: (a) $Co_{72}Cr_{28}$, and (b) $Co_{63}Cr_{28}Ta_9$.The histograms were fitted using multiple Gaussian components.

.

[1] NanoScope Analysis v1.40r1 Now Availiable - Free Download - The Nanoscale World, (n.d.). http://nanoscaleworld.bruker-axs.com/nanoscaleworld/forums/t/812.aspx (accessed May 25, 2025).

[2] R. Tran, X.-G. Li, J.H. Montoya, D. Winston, K.A. Persson, S.P. Ong, Anisotropic work function of elemental crystals, Surface Science 687 (2019) 48–55. https://doi.org/10.1016/j.susc.2019.05.002.

[3] A. Kiejna, K.F. Wojciechowski, Work function of metals: Relation between theory and experiment, Progress in Surface Science 11 (1981) 293–338. https://doi.org/10.1016/0079-6816(81)90003-4.

2. Gaussian fitting parameters for the work-function histograms obtained from the KPFM potential maps

Table S1. Gaussian fitting parameters obtained from KPFM work function distributions.

Alloy	Gaussian peak	Center (eV)	FWHM (eV)	Peak-center range (eV)	R^2	Main interpretation
$Co_{72}Cr_{28}$	I	3.94	0.28	0.00	0.995	Unimodal, electronically continuous
$Co_{69}Cr_{28}Ta_3$	I	4.13	0.12	0.49	0.992	Multimodal, distinct electronic subpopulations
	II	4.52	0.09			
	III	4.62	0.12			
$Co_{66}Cr_{28}Ta_6$	I	4.15	0.32	0.00	0.972	Unimodal, electronically continuous despite local contrast
$Co_{63}Cr_{28}Ta_9$	I	3.83	0.29	0.88	0.996	Strongly multimodal and heterogeneous
	II	4.15	0.38			
	III	4.71	0.62			

3. Spatial distribution of the Co-Cr matrix and $TaCO_2$ precipitates – SEM

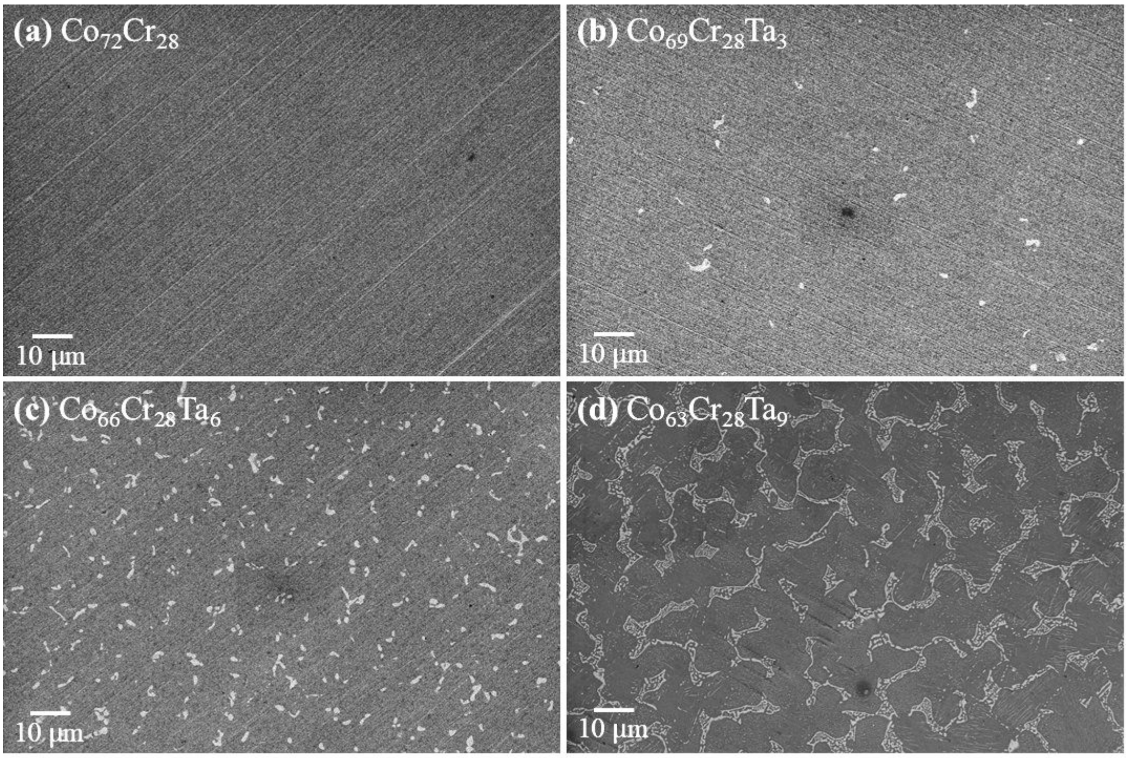

Figure S5. Surface microstructure image of (a) $Co_{72}Cr_{28}$, (b) $Co_{69}Cr_{28}Ta_3$, (c) $Co_{66}Cr_{28}Ta_6$, and (d) $Co_{63}Cr_{28}Ta_9$.

4. Quadratic relationship between the apparent area fraction $TaCo_2$ and Vickers microhardness, contact potential difference, and contact angle

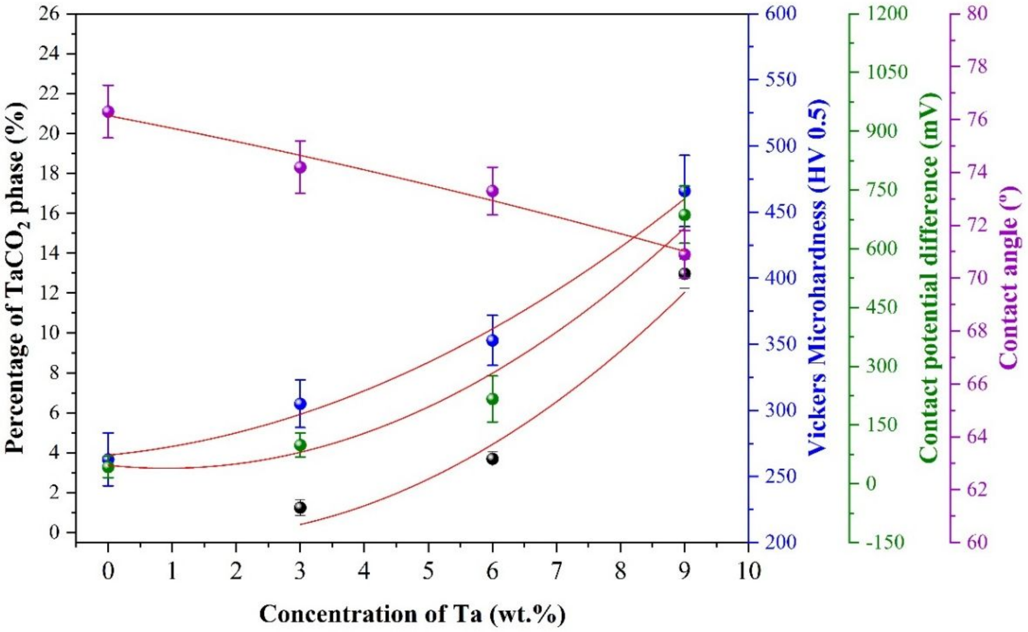

Figure S6. Comparison between the percentage of $TaCo_2$ phase and the Vickers microhardness, contact potential difference and contact angle properties for the samples $Co_{72}Cr_{28}$, $Co_{69}Cr_{28}Ta_3$, $Co_{66}Cr_{28}Ta_6$ and $Co_{63}Cr_{28}Ta_9$. The spheres indicate the average value, the red line indicates the polynomial fit and the bars are the standard deviations.

Table S2. Description of the polynomial adjustments performed on the sample data as a function of Ta concentration (0, 3, 6 and 9 wt. %).

Property	Polynomial fit of property (y) as a function of Ta concentration (x)	R^2
Percentage of $TaCo_2$ phase (%)	$y = -0.46x + 0.2x^2$	0.98
Vickers Microhardness (HV0.5)	$y = 227 + 5x + 2x^2$	0.99
Contact potential difference (mV)	$y = 48 – 17x + 9x^2$	0.98
Contact angle (º)	$y = 76 – 0.35x – 0.03x^2$	0.98
